# Health Tract, No. 270

**Published:** 1867-01

**Authors:** 


HEALTH TRACT, No. 270.

### CAUSE OF AGUE.

A correspondent of the *Prairie Farmer* says that he was prevented for ten years from emigrating to Illinois by the apprehension of suffering from the prevalent sickness of the country; and expressed the opinion that thousands of others spend year after year in listless inactivity, or in the comparatively profitless cultivation of the stoney soil of the East, when they might soon become independent, thriving farmers in the boundless West, where there is a fine, rich soil, a mild climate and a plenty of room. He observes that the people were sickly where he was "raised," until they derived their family supplies of water from well cemented cisterns; by which he probably meant, that if rain-water was used for all cooking and drinking purposes, fever and ague, with many kindred ailments would disappear.

There is fever and ague in the South, and plenty of it, in its most aggravated form; and yet, in cities, villages and on the plantations, cistern-water, obtained from the roofs of buildings, is very generally used. There is more or less of chill and fever in the torrid and temperate zones, whether in the old world or the new. The presumption is, that as people live to a good old age in all latitudes, the water of each country is adapted to the health of that country. The earth was certainly intended to be cultivated and replenished; to be filled with thriving people.

Wrong practices follow wrong theories; hence it is important to understand the true cause of fever and ague. As the malady prevails only in warm weather, and does so within the antartic circles, it must arise from something invariably connected with these latitudes; and that thing seems to be, as far as our present knowledge extends, the combination of three elements, heat, moisture and vegetable product. These three ensure one result, vegetable decomposition, giving rise to a constituent of the atmosphere of that locality, which originates that disease known as fever and ague and its kindred maladies, epidemic diarrhea and fevers. Whether this constituent is inert matter, or possesses vegetable life, or is of a breathing animal nature, the laws by which it is generated are one and the same; and there are two ways of successfully contending with it:—to prevent its formation by proper drainage of the face of the country, or to resist its pernicious influences by keeping fires in our sitting-apartments for the hour including sunrise and sunset, these being the times when the atmosphere is known to be most loaded with the offending ingredient, which is thoroughly expurged by a sufficient amount of heat.—*Watchman and Reflector.*

